# Mixing Regime Controlled Kinetics of Amorphous Calcium Phosphate Formation and Transformation

**DOI:** 10.1021/acsomega.6c01349

**Published:** 2026-06-12

**Authors:** Özgür Gülmez, Eren Demirbilek, Seniz Ucar

**Affiliations:** † Middle East Technical University, Department of Metallurgical and Materials Engineering, Üniversiteler Mahallesi, Dumlupınar Bulvarı No:1, 06800 Ankara, Turkiye; ‡ 8018 Norwegian University of Science and Technology, Department of Chemical Engineering, Sem Sælands vei 4, 7491 Trondheim, Norway

## Abstract

Calcium phosphate precipitation plays a central role in biomineralization processes and the design of biomaterials, with amorphous calcium phosphate (ACP) often acting as a transient precursor to more stable crystalline phases such as hydroxyapatite (HA). Despite extensive research, the mechanistic pathways of ACP formation and transformation remain only partially understood, as they are highly system-specific and sensitive to dynamic changes in solution chemistry. Here, in situ potentiometric monitoring, combining pH and calcium ion-selective electrode (Ca-ISE) measurements, is used to monitor ACP precipitation and transformation under different mixing regimes and in the presence of model additives. Complementary solid-phase characterization is employed to validate phase identity and composition. All systems followed a two-step ACP-mediated pathway, characterized by an initial metastable period followed by ACP precipitation and subsequent transformation to poorly crystalline HA. However, significant differences in kinetics were observed: the *P in Ca* setup exhibited delayed ACP nucleation and extended ACP lifetime compared to *Ca in P*, while additive presence further prolonged ACP stability, with citrate exerting a stronger effect than BSA. These variations were linked to differences in pH evolution, transient supersaturation, and solution speciation established during precursor mixing. Our findings highlight in situ potentiometric measurements as a valuable, accessible approach for probing calcium phosphate crystallization, provided its limitations are recognized and interpretations are supported by complementary characterization methods.

## Graphical abstract

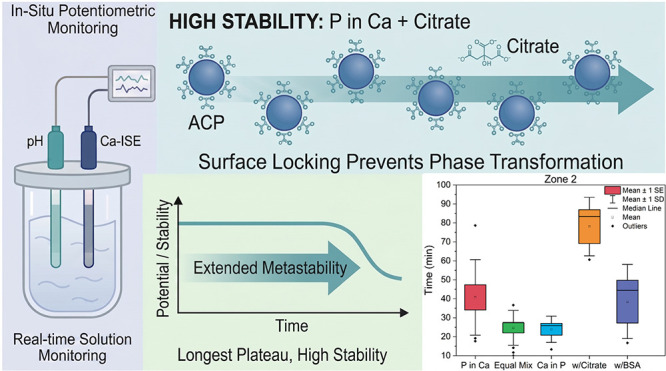

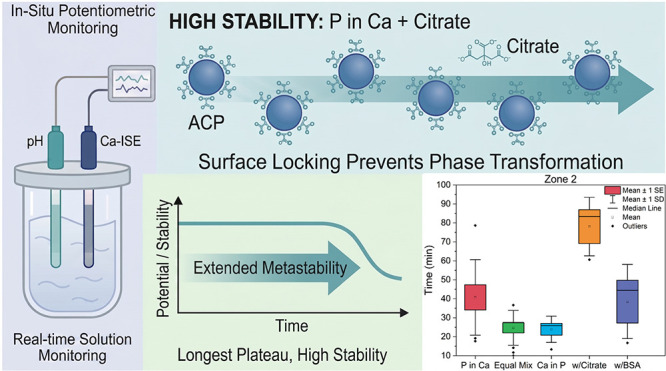

## Introduction

Calcium phosphate (CaP) phases are ubiquitous in biological systems and play a central role in biomineralization, tissue engineering, and biomaterials science. Among these phases, amorphous calcium phosphate (ACP) has drawn significant attention due to its role as a metastable precursor to crystalline forms such as octacalcium phosphate (OCP), tricalcium phosphate (TCP), and hydroxyapatite (HA); the principal inorganic component of bone and teeth.^1,2^ The significance of ACP lies not only in its transitional nature but also in its functional tunability for biomedical applications such as drug delivery, bone grafts, and remineralization agents.^3,4^ A critical challenge for its practical utilization lies in controlling its transformation behavior.

From a thermodynamic perspective, ACP formation and transformation align with Ostwald’s rule of stages, which suggests that systems may evolve through a series of metastable states before reaching the most stable crystalline phase.^5^ Owing to their higher solubility and lower activation energy barriers, amorphous phases such as ACP form rapidly, but are eventually replaced by more stable phases, driven by the thermodynamic imperative to minimize free energy.^6–8^ The formation of ACP and transformation from ACP to crystalline phases is a complex, multistep process with significant implications for both biological mineralization and the design of ACP-based biomaterials.

Early investigations into ACP formation and transformation relied heavily on ex situ characterization, which provided valuable kinetic data but limited mechanistic insight due to the inability to capture transient intermediates.^9,10^ The emergence of advanced analytical techniques with improved spatiotemporal resolution has enabled detailed investigations of ACP formation and transformation under in situ conditions, offering unprecedented insight into its dynamic nature, and stimulating ongoing scientific debates on classical vs nonclassical pathways. Recent studies have employed time-resolved X-ray diffraction, Raman spectroscopy, and cryogenic or liquid-cell transmission electron microscopy (TEM) to capture the dynamic evolution of ACP into crystalline calcium phosphates in real time.^7,11–16^ Furthermore, synchrotron-based in situ small- and wide-angle X-ray scattering (SAXS/WAXS) has proven invaluable for elucidating nanoscale structural changes during phase evolution.^17,18^ These advanced in situ techniques have provided insights into ACP formation and transformation pathways that were previously inaccessible; however, they also come with limitations. Access to high-end instrumentation, particularly synchrotron sources, is scarce, and some methods require working with small sample volumes, introducing confinement effects. Additionally, their applicability is often limited to specific supersaturation and time ranges, which may not fully replicate conditions encountered in natural or synthetic systems.

Among the experimental techniques available for real-time monitoring of amorphous phase formation and subsequent phase transformations, in situ potentiometric monitoring stands out as a versatile and informative method for investigating multistep crystallization pathways and reaction kinetics.^10^ First applied to calcium carbonate systems, this approach involves the gradual titration of calcium into a buffered carbonate or phosphate solution under constant pH conditions, while continuously monitoring calcium activity with a calcium ion-selective electrode (Ca-ISE) in most of the studies.^19,20^ This configuration enables precise tracking of time-dependent kinetics and assessment of how experimental conditions and additives influence reaction rates. Furthermore, when calcium activity data are combined with carbonate or phosphate speciation calculations, it becomes possible to determine the evolving stoichiometry of forming species, whether in the form of solution clusters or solid precipitates, at each stage of the reaction.^16,21^ This capability allows the allocation of distinct stages in the reaction pathway and provides mechanistic insight into both phase formation and transformation processes.^22^ The synergistic effects of present additive type and timing of addition on ACP crystallization kinetics has also been investigated by integrating in situ pH and turbidity measurements with solid-phase characterization, where additives with weak interactions with ACP were shown to either inhibit or accelerate crystallization depending on their addition before or after ACP formation.^23^ In our previous work, potentiometric titration was used to generate metastable supersaturated solutions, followed by simultaneous monitoring of pH and calcium activity to capture the dynamics of amorphous calcium phosphate (ACP) formation and transformation.^8^ This method proved effective for identifying key transition points during ACP evolution and elucidating the role of additives in modulating solution chemistry and crystallization kinetics.

Given the importance of understanding ACP formation and transformation kinetics and tuning it for the design of bioactive ceramics, drug delivery carriers, and regenerative scaffolds, this study systematically investigates the effects of precursor mixing conditions and additive presence on ACP formation and stability, via coupling of solution chemistry monitoring and characterization of precipitates. While in situ potentiometric approaches have previously been applied to investigate ACP formation and transformation, studies using different sequential mixing approaches, where precipitation occurs during precursor addition, have demonstrated that precipitation pathways can be altered due to variations in transient supersaturation and pH at the onset of precipitation, the present study revisits this methodology.^24^ Specifically, it aims to decouple the effects of precursor mixing regime from the final solution composition, thereby enabling a direct assessment of how time-resolved supersaturation and speciation, established during mixing and prior to precipitation, govern ACP nucleation kinetics and stability. The sensitivity of ACP to its surrounding environment can make the initial precipitation conditions critical in directing amorphous-mediated crystallization pathways. In particular, the mixing regime of calcium and phosphate precursor solutions alters the time-dependent supersaturation and ionic speciation. Additionally, we examine the role of two representative additives: citrate, a tricarboxylate metabolite abundant in bone that can specifically interact with calcium phosphate surfaces to delay crystallization; and bovine serum albumin (BSA), a model macromolecule known to adsorb nonspecifically onto mineral phases.^25–27^ By comparing these additives, the influence of molecular specificity and size on ACP formation and stability is shown via in situ investigations.

## Materials and Methods

### Materials

Calcium nitrate tetrahydrate (Ca­(NO_3_)_2_·4H_2_O) and potassium hydroxide (KOH) were purchased from Merck. Trisodium citrate dihydrate (Na_3_C_6_H_5_O_7_·2H_2_O) and bovine serum albumin (BSA) were purchased from Sigma. Potassium dihydrogen phosphate (KH_2_PO_4_) and potassium nitrate (KNO_3_) were purchased from Isolab. All solutions were prepared with ultrapure deionized water (DIW) with a conductivity of 0.055 μS cm^–1^.

## Methods

All experiments were conducted in a 0.5 L double-walled glass reactor and two baffles were attached to the lid. Temperature was controlled with a DC-2006 circulating water bath at 25 °C. Reactor content was stirred with a magnetic stirrer at 500 rpm throughout the experiments. Nitrogen, saturated with water, was continuously bubbled in the reaction vessel to introduce an inert atmosphere to isolate the reaction environment from air intrusion and prevent carbon dioxide dissolution. The pH was recorded continuously by means of a combined glass electrode with a KCl reference electrolyte and calcium ion activity in the vessel was measured in situ by a calcium ion specific electrode (Ca-ISE), both connected to the Metrohm 907 Titrando setup. Both electrodes were calibrated daily (see Supporting Information, Section A, Figure S1). The chemical speciation, calcium ion activity and activity-based supersaturation index (SI) were determined by the thermodynamic calculation program Minteq 3.0 (KTH, Royal Institute of Technology, Stockholm, Sweden), according to eq 1, using the Minteq v4 database and Davies model with a *b* parameter of 0.3. The ionic activity products (IAP) were calculated by multiplication of activities of each ion forming the corresponding solid phases. The equilibrium constants and solubility products, *K*_sp_, used for calculations are given in the Supporting Information, Section B.

1 $$\mathrm{SI}=\log\left(\frac{\mathrm{IAP}}{K_{\mathrm{sp}}}\right)$$

Spontaneous precipitation of calcium phosphates was carried out in batch experiments by preparing supersaturated metastable solutions and allowing the precipitation to occur. The initial metastable solutions were prepared using three different mixing procedures. In the first setup, referred to as *Ca in P*, a 500 mL phosphate solution (2.4 mM KH_2_PO_4_) containing 50 mM of KNO_3_ and 1.92 mM of KOH was prepared. KNO_3_ was used to maintain constant ionic strength throughout the experiments and KOH was used for pH adjustment. Then, 40 mL of 50 mM Ca­(NO_3_)_2_·4H_2_O was added to the phosphate solution with a 2 mL min^–1^ flow rate. In the second setup, referred to as *P in Ca*, a 40 mL solution consisting of 24 mM KOH and 30 mM KH_2_PO_4_ was added to the reactor, initially containing a 500 mL solution of 50 mM KNO3 and 4 mM Ca­(NO_3_)_2_·4H_2_O, at a 2 mL min^–1^ flow rate. In the third setup, referred to as *Equal Mix*, a 20 mL solution of 48 mM KOH and 60 mM KH_2_PO_4_; and 20 mL 100 mM Ca­(NO_3_)_2_·4H_2_O solution were added simultaneously in the reactor, containing 500 mL 50 mM KNO_3_ solution, with a flow rate of 1 mL min^–1^ for each solution. Depending on the specific mixing procedures and the corresponding starting solution, the initial pH values measured in the reactor varied. The phosphate precursor used in the *Ca in P* setup had starting pH values changing between 7.46 to 7.52, whereas, the initial solutions for the *P in Ca* and *Equal Mix* setups exhibited initial pH values between 6.0 and 6.5. Addition of solutions at a fixed rate was performed using a Metrohm Dosino 800 automatic titrator device for all setups. In all experimental sets the initial metastable solution after complete mixing had the same composition prior to precipitation (see Supporting Information, Section C, Table S1).

In order to investigate the effects of additives, citrate or BSA was introduced into the initial precursor solutions at a concentration of 5 ppm prior to the addition of the second precursor. Specifically, these experiments were performed using sequential mixing regimes; both citrate and BSA presence were evaluated in the *Ca in P* setup, while citrate was additionally tested in the *P in Ca* setup. The precipitates were collected at different reaction stages via vacuum filtration, washed with DIW and ethanol, and left to dry under atmospheric conditions. To ensure the reliability of the kinetic observations and accurately capture the systems’ behavior, multiple independent replicates were performed for each experimental setup. The number of independent replicates (*n*) ranged from 3 to 12 depending on the specific mixing regime. Specifically, the simultaneous *Equal Mix* setup, being most prone to fluctuations, was replicated up to 12 times to robustly establish its baseline kinetic profile. For the sequential addition setups and the additive experiments, the number of independent replicates ranged between 3 and 9. The pH data from all experiments can be found in Supporting Information, Section D, Figure S2.

Precipitates collected at different time points of the experiments were imaged by scanning electron microscopy (SEM, FEI Nova Nano 430). Samples were sputter-coated with gold prior to imaging and an accelerating voltage of 20 kV was used. Transmission electron microscopy (TEM) was performed using a JEOL JEM-2100F microscope equipped with a Schottky-type field emission gun (FEG) at an accelerating voltage of 200 kV. Samples were prepared by drop-casting onto carbon coated copper grids at various time points during the reactions. Phase analysis of collected samples were conducted via powder X-ray diffraction (XRD, Bruker D8 Advance) in the 2θ range of 10°-75° with a step size of 0.0205° and a step time of 2.3 s. Samples were ground in a mortar prior to measurements to prevent any coagulation between particles, which deflects incident X-rays to unwanted angles. Raman microspectroscopy (Renishaw, InVia Reflex) was used with a 532 nm 100 mW Nd:YAG laser through a 50x lens and at a laser power of 10% to prevent laser-induced phase transformations. Fourier transform infrared (FTIR, Shimadzu IRTracer-100) spectra were collected in mid- IR region 4000–500 cm^–1^. Inductively Coupled Plasma Optical Emission spectroscopy (ICP-OES, PerkinElmer Optima 4300DV) was used to determine the Ca/P ratios of precipitates. For this purpose, approximately 5 mg of each respective solid sample was dissolved in 10 mL of 1 M HCl, and the resulting solution was diluted to a final volume of 50 mL using DIW. Thermogravimetric analysis (TGA, SII NanoTechnology, EXSTAR 7300) was conducted on samples collected at the first pH plateau, between 30 and 900 °C, with a heating rate of 10 °C min^–1^ under N_2_ atmosphere, and the derivative thermogravimetry curve (DTG) was obtained from the derivative of mass change over time as a function of temperature. Zeta potential measurements of the particles collected at the first pH plateau and at the end of experiments were performed by using folded capillary cells at a 50 V effective voltage with 5 measurements each averaged over 100 runs (Malvern Zetasizer Ultra). The Von Smoluchowski equation was applied for calculations. Measurements were conducted in 10 mM Tris buffer solution at a pH of 7.4 and 10 mM KNO_3_ was used as an ionic strength adjuster. Samples were collected from the reactor via filtration at designated time points and redispersed in the buffer solution via sonication prior to measurements.

## Results and Discussion

In order to elucidate the parameters influencing ACP formation and stability, experiments were conducted using three different mixing procedures and two distinct additives. The final solution composition upon complete mixing was identical in all experiments and was based on conditions previously identified to promote a two-step, ACP-mediated hydroxyapatite precipitation mechanism.^8^ In situ pH measurements recorded from the moment of complete mixing revealed similar trends across all conditions, characterized by an initial metastable supersaturated period followed by two distinct pH drops (Figure 1A). These zones are marked for a representative *Ca in P* setup in Figure 1B. Zone 1 represents the 20 min mixing period of precursor solutions for all experiments.

**Figure 1:**
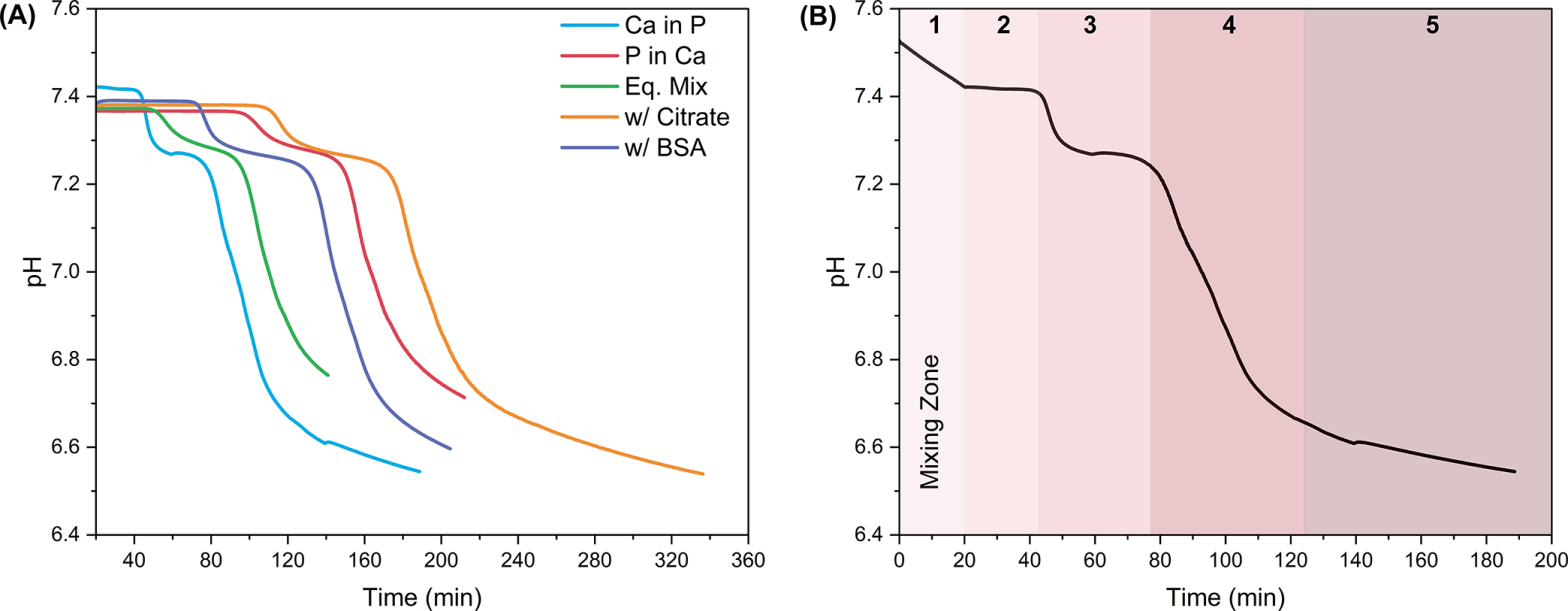
(A) Representative in situ pH profiles recorded as a function of time during spontaneous precipitation reactions for the specified mixing setups and for additive-containing conditions in the *Ca in P* setup. The initial time point at 20 min corresponds to the completion of solution mixing. (B) A representative pH profile from an additive-free *Ca in P* experiment, divided into five zones based on distinct changes observed in the signal.

At the end of mixing, a metastable supersaturated solution is obtained and its lifetime is given by Zone 2. The supersaturation indices at this zone are calculated to be 12.5 and 3.07 for hydroxyapatite (HA) and amorphous calcium phosphate (ACP), respectively. In Zone 3, a noticeable pH drop occurs, followed by a second stabilization. At this stage, the solution transitions from transparent to slightly turbid, indicating the onset of precipitation, which was operationally defined as the exact point where the pH signal exhibited a 5% decrease of its total step height (determined via the Fall Time gadget in OriginPro). Intermediate samples were collected from this region for characterization. The XRD patterns of precipitates collected in Zone 3 exhibit broad, diffuse features, without any sharp diffraction peaks as an indication of their amorphous nature (Figure 2A). The presence of ACP was further confirmed using FTIR and μ-Raman spectroscopy (Figure 2B–C). FTIR spectra of the intermediate samples show strong absorption bands at ∼550 cm^–1^ and ∼1000 cm^–1^, corresponding to the ν_4_ and ν_3_ bending modes of PO_4_^3–^, respectively. A broad peak at ∼3200 cm^–1^ indicates water content, which is typically high in ACP structures (see Supporting Information, Section E, Figure S3).^28^ Raman spectra show broad bands around at 950 cm^–1^. Despite the higher driving force for HA formation, precipitation is governed more strongly by kinetic factors than by thermodynamic stability at this stage.^29^ As a metastable phase, ACP forms preferentially due to its lower nucleation barrier, while the formation of HA is kinetically hindered. Zone 3, corresponding to the lifetime of ACP, concludes with a second sharp pH decrease, signifying a subsequent precipitation event. Zone 4 is defined as the duration of the phase transformation process. Final precipitates were collected for characterization at the end of Zone 5, which begins at the intersection point of tangent lines drawn on the pH curve at Zone 4 and the final region exhibiting a relatively slow pH decrease. XRD patterns of the final precipitates confirmed the presence of poorly crystalline HA (Figure 2A). Additional low-angle measurements were performed to verify the absence of octacalcium phosphate (OCP) in the final products, as this phase exhibits a distinctive peak at 4.7° 2θ (see Supporting Information, Section F, Figure S4). FTIR spectra of the final samples show distinct ν_4_ and ν_3_ bending modes of PO_4_^3–^ at ∼557 cm^–1^ and ∼1000 cm^–1^. Importantly, a doublet feature appears near 600 cm^–1^, one of the key spectral indicators distinguishing HA from ACP (Figure 2B).^30^ μ-Raman spectra of the final samples display a strong, sharper ν_1_ PO_4_^3–^ symmetric stretching band at 960 cm^–1^ (Figure 2C).^31^ Additional characterization of precipitates collected at Zone 3 via electron microscopy imaging and electron diffraction revealed ACP particles with diameters of approximately 30–50 nm. The absence of diffraction spots at this stage confirmed their amorphous nature, while the emergence of diffraction features from Zone 4 onward indicates the onset of crystallization. These observations are consistent with, and support, the two-step precipitation pathway identified through potentiometric measurements (see Supporting Information, Section G, Figure S5).

**Figure 2:**
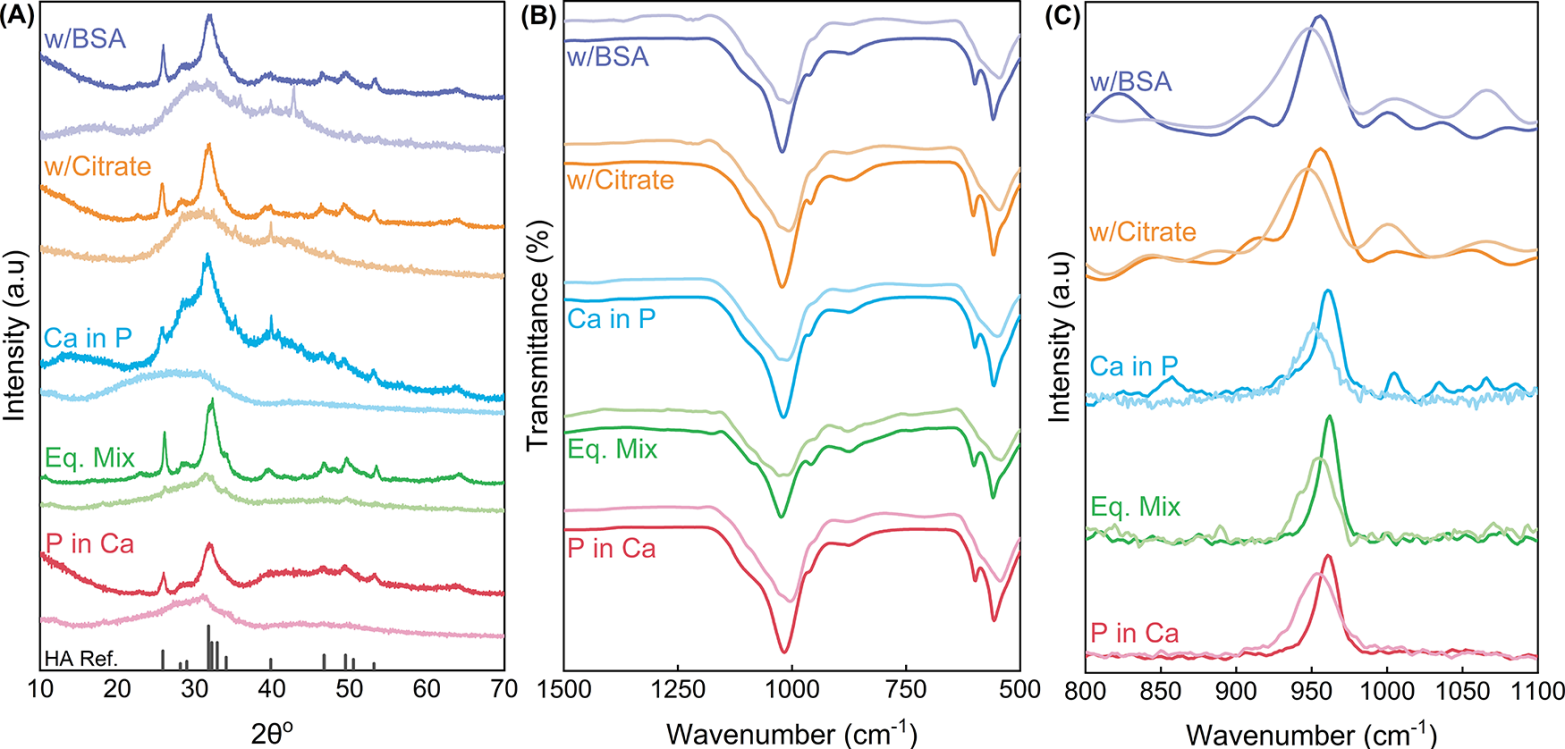
(A) XRD, (B) FTIR, and (C) μ-Raman spectra of precipitates collected during Zone 3 and Zone 5 for the specified mixing setups and for additive-containing conditions in the *Ca in P* setup. The light and dark colored curves represent Zone 3 and Zone 5, respectively for each experimental setup.

Although all experimental conditions followed a similar ACP-mediated pathway for HA formation, the reaction kinetics varied among the setups (Figure 3). While variations between experimental conditions are evident, particularly for Zone 2 and Zone 3, these trends are interpreted in conjunction with the observed distributions and reproducibility of the data sets. The *Ca in P* and *Equal Mix* configurations exhibited comparable average durations across all reaction zones. In contrast, the *P in Ca* setup showed extended times in Zones 2 and 3, suggesting delayed ACP precipitation and enhanced ACP stability. This setup also exhibited a greater number of outliers in Zone 2 compared to the *Ca in P* configuration. Both additives delayed ACP formation and prolonged its lifetime, with citrate exerting a more pronounced effect. Furthermore, in the presence of citrate, phase transformation concluded over a longer time scale as seen via Zone 4.

**Figure 3:**
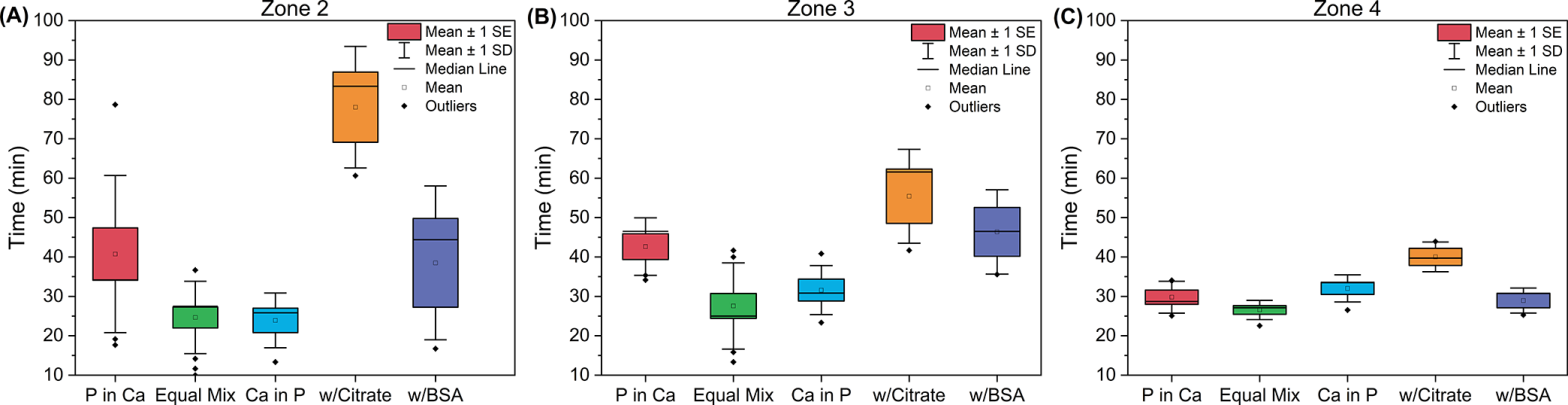
Duration of (A) Zone 2, (B) Zone 3, and (C) Zone 4 for the specified mixing setups and for additive-containing conditions in the *Ca in P* setup. Data are presented as box plots illustrating the distribution, median, and spread of replicate measurements.

To elucidate the differences observed in ACP precipitation kinetics, the solution chemistry during the establishment of supersaturation was investigated using detailed in situ measurements of pH and calcium activity, complemented by thermodynamic modeling. The thermodynamic calculations of solution speciation and pH during precursor mixing are given in Supporting Information, Section H1 for all experimental setups as a function of time (Figures S6–S8). These distinct pH profiles reflect differences in phosphate speciation and ion-pair formation dynamics during precursor addition. Figure 4A presents the experimental pH evolution during precursor mixing for each setup. In all experimental configurations, regardless of additive presence, the measured pH profiles closely matched those calculated thermodynamically (Figure S6). For the *Ca in P* configurations, a nearly linear decrease from 7.50 to 7.40 was observed upon calcium solution addition. Conversely, the *P in Ca* and *Equal Mix* showed an immediate increase in pH upon mixing, primarily due to the low buffering capacities of the solutions initially present in the reaction medium. The pronounced run-to-run variability in ACP nucleation kinetics and the duration of Zone 2 observed specifically within the *P in Ca* setup was directly attributed to fluctuations in solution pH during precursor mixing. Owing to the low buffering capacity of the calcium solution, these fluctuations resulted in final pH values ranging from 7.44 to 7.32. Importantly, the duration of Zone 2 was closely correlated with the final pH, with lower pH values leading to further delays in nucleation. This trend aligns with the reduced supersaturation with respect to ACP under lower pH values, demonstrating the significant role of the thermodynamic driving force on the variability of nucleation kinetics within this specific setup (see Supporting Information, section I, Figure S9)., In contrast, the other setups exhibit considerably smaller fluctuations in the final pH upon completion of precursor addition, indicating comparable supersaturation conditions (see Supporting Information, Section D, Figure S2).

**Figure 4:**
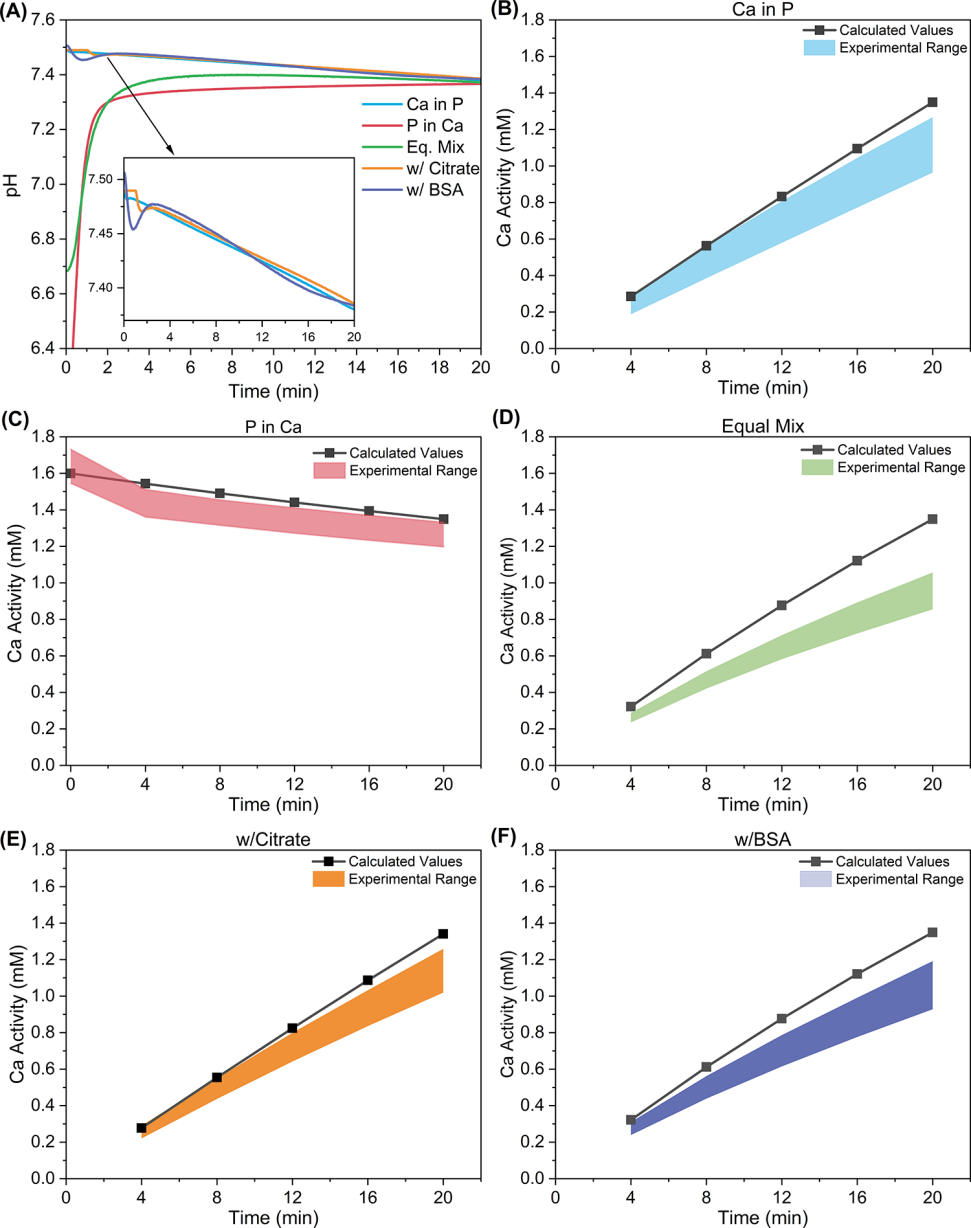
(A) In situ pH profiles recorded during Zone 1 while mixing precursor solutions for the specified mixing setups and for additive-containing conditions in the *Ca in P* setup. (B–F) Comparison of theoretically calculated and experimentally measured calcium activities under the specified experimental conditions during Zone 1.

Figure 4(B–F) presents both the theoretically calculated and experimentally measured calcium ion activity profiles recorded during precursor mixing for all experimental setups (see Supporting Information, Section H2). In additive-free experiments, the *Equal Mix* setup exhibited the largest deviations from theoretical predictions, both in terms of absolute calcium activity values and the linearity of its profile. Due to the simultaneous addition of both precursor solutions, fluctuations in calcium activity were the greatest under this condition. Compared with the *Ca in P* setup, ACP precipitation was noticeably delayed in the *P in Ca* setup and in the presence of additives, most drastically with citrate. Despite these kinetic differences, the experimental calcium activity profiles showed similar overall behaviors in these systems, when compared to the theoretical values, maintaining comparable magnitudes and near-linear trends over time. The observed near-linear calcium activity profiles are consistent with thermodynamic calculations that assume predominant formation of ion-association complexes containing single calcium ions.^32^ It should be noted, however, that Ca-ISE measurements provide an indirect probe of solution speciation and do not directly resolve the size or structure of transient ion clusters. The lower experimentally measured calcium activity values compared to equilibrium-based theoretical predictions may reflect the presence of transient ion pairs and higher-order associations formed under the dynamic, nonequilibrium conditions of precursor mixing.^16^ Therefore, while the agreement between experimental data and theoretical trends is consistent with the predominance of single-ion pairing, the coexistence of small, dynamic prenucleation assemblies cannot be excluded. These findings suggest that the overall delays in ACP formation between different mixing routes likely arise from kinetic limitations, hindering effective collisions and/or preventing ion-clusters from reaching critical size, rather than from thermodynamic constraints on phase separation such as lowered supersaturation with respect to ACP. Similar kinetic interpretations have been reported for ACP formation in the presence of additives, where precipitation was delayed even at concentrations insufficient to alter the effective supersaturation of the solution.^8,30^ The more pronounced delay observed with citrate compared to BSA can be attributed to the substantially higher molar concentration of citrate at the same mass concentration (5 ppm), due to its lower molecular weight. Furthermore, at the experimental pH range, citrate’s carboxyl groups are deprotonated and negatively charged, promoting strong electrostatic interactions with calcium ions. Contrary to our deductions, in a previous work Ruiz-Agudo et al., proposed that citrate delays early ACP formation by the phase separation and stabilization of a liquid-like precursor. However, it is important to note that the citrate-to-calcium molar ratio in the referred study was up to 20 times higher than in the present work, representing a significantly different solution environment.^33^

In additive-free conditions, the observed kinetic barriers likely arise from differences in solution speciation during precursor mixing, despite identical final solution compositions across all setups. Theoretical calculations (see Supporting Information, Section H1) reveal that at early time points, in the P in Ca setup, calcium ion (Ca^2+^) concentrations are higher, while the concentrations of the dominant phosphate species (HPO_4_^2–^ and H_2_PO_4_^–^) are lower compared to the Ca in P setup; the concentrations of calcium–phosphate ion pairs are, however, comparable in both configurations. In order to elucidate the role of solution species in ACP precipitation kinetics, Turhan et al. investigated the early stages of ACP formation using high temporal resolution techniques, including NMR, turbidimetry, SAXS, cryo-TEM, and calcium potentiometry.^17^ Their findings indicated the formation of transient prenucleation clusters; however, ACP nucleation were not found to necessarily proceed via aggregation or dehydration of these clusters. Instead, nucleation was shown to occur through a dissolution–reassembly mechanism. These results are in agreement with the work of Habraken et al., where the prenucleation clusters are identified as soluble ion-association complexes of [Ca­(HPO_4_)_3_]^4–^, which subsequently take up calcium ions to form insoluble postnucleation clusters, [Ca_2_(HPO_4_)_3_]^2–^, that precipitate as ACP.^16^ These studies align with our observations, which suggest that simple ion association complexes primarily form during precursor mixing. In addition, the calcium triphosphate stoichiometry reported for such species provides a plausible rationale for prolonged precipitation times in the *P in Ca* setup, where phosphate availability is the limiting factor.

The duration of Zone 3 represents the lifetime of amorphous calcium phosphate (ACP) in solution, defined as the time it remains in its amorphous state before crystallization, and can vary from seconds to hours depending on environmental conditions such as ion concentrations and molar ratios of calcium and phosphate, pH, temperature, ionic strength, and the presence of stabilizing agents.^34–37^ Across all experimental conditions, poorly crystalline hydroxyapatite (HA) consistently formed as the subsequent crystalline phase, making the length of Zone 3 a valuable indirect indicator of HA nucleation and early growth kinetics.

In the absence of additives, the ACP lifetime was longer in the *P in Ca* setup than in the *Ca in P* setup, and both additives extended the stability of ACP when introduced in the *Ca in P* setup. Additional experiments were carried out by addition of citrate in *P in Ca* setup, where a similar profile of extended ACP lifetime was observed (see Supporting Information, Section J, Figure S10). Previous work revealed that ACP precipitates can be denoted by the formula, Ca_*x*_H_*y*_(PO_4_)_*z*_·*n*H_2_O, where the amount of water and the calcium to phosphate molar ratio (Ca/P) vary depending on the synthesis conditions, and more acidic forms obtained under lower pH with higher amounts of [HPO_4_]^2–^ are less stable toward transformation into HA.^3^ In all setups, ACP precipitation occurred only after complete mixing of the precursor solutions, under identical solution conditions, however we hypothesized that the differences in time-resolved solution speciation and interaction of additives with precursor ions can lead to compositional differences in the forming ACP. In order to investigate such differences, investigations on Ca/P ratios, thermogravimetric analyses, and zeta potential measurements were conducted on ACP phases (Figure 5). The Ca/P ratios determined via ICP-OES were similar within experimental uncertainty for all samples (see Supporting Information, Section K, Figure S11). It should be noted, however, that due to its highly metastable and heavily hydrated nature, the ACP structure can easily change (e.g., via hydration loss or localized transient phase changes) during particle isolation and drying prior to analysis; thus, these ex situ compositional results must be interpreted with caution.^38^

**Figure 5:**
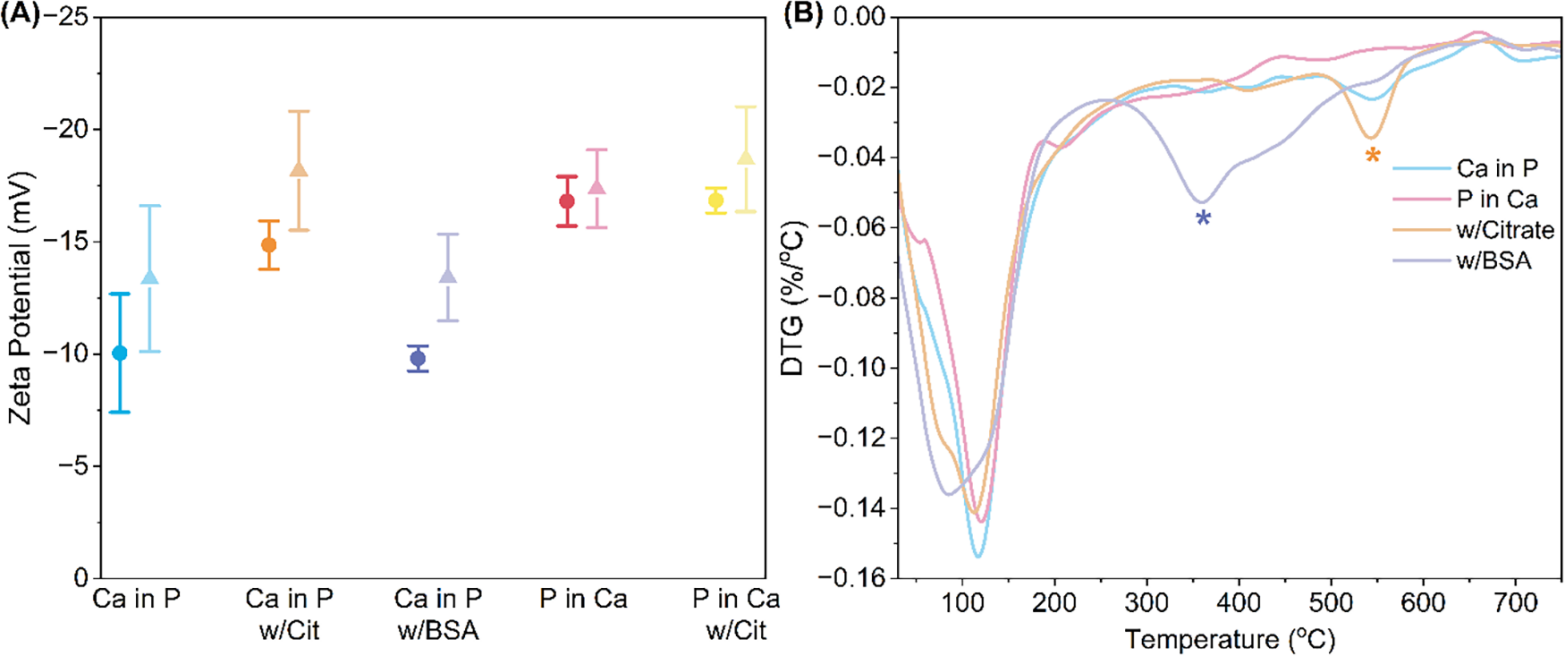
(A) Zeta potential values of particles collected during Zone 3 (shown with triangles) and Zone 5 (shown with circles) under the specified experimental conditions, (B) Derivative thermogravimetric (DTG) curves, showing BSA and citrate presence in precipitates obtained in *Ca in P* setup via mass loss around 350 and 500 °C, respectively (marked with *).

Zeta potential measurements of ACP particles showed that the magnitude and persistence of the negative surface charge increased in the *P in Ca* setup and upon citrate addition in both mixing setups (Figure 5A). These values are consistent with reported zeta potential values of BSA (approximately −25 mV) at pH 7.4.^39^ This behavior coincides with the extended ACP stability and the delayed transition into Zone 4. In contrast, the presence of BSA resulted in smaller and less pronounced changes in zeta potential, consistent with its comparatively weaker influence on ACP stability. Because zeta potential measurements probe the surface properties of the particles, the measured values are regarded as surface-chemical fingerprints that reflect differences in surface termination, hydration state, and additive association established during ACP formation and manifested during Zones 3 and 4. Within this framework, the enhanced stability of ACP observed for the *P in Ca* route, particularly in the presence of citrate, is consistent, indicating increased electrostatic stabilization of the amorphous phase likely driven by the surface adsorption of the additives.

As detailed in Supporting Information H2, calculations demonstrate that citrate addition has a negligible effect on the free calcium activity and supersaturation, effectively ruling out bulk solution complexation as the primary stabilizing factor. A widely accepted hypothesis for additive-induced stabilization of ACP is that additives suppress the heterogeneous nucleation of more stable phases, where ACP is utilized as a substrate.^30^ The comparatively stronger stabilization effect observed with citrate is thus attributed to its higher molar concentration relative to BSA and its known specific affinity for calcium ions, which would enhance its surface interaction with ACP. Thermogravimetric analyses of ACP samples formed in the presence of additives revealed additive-related mass losses, with corresponding peaks clearly observed in the derivative thermogravimetric (DTG) curves. These findings indicate surface adsorption as the dominant mechanism, suggesting that the suppression of heterogeneous nucleation is a probable consequence of the adsorbed molecules physically blocking the active sites on the ACP surface, thereby lending support to this hypothesis in the present study (Figure 5B).

A defining feature of ACP is its responsiveness to molecular additives, especially polymeric and biomolecular species that interact with its surface or bulk structure. Organic molecules, particularly polyelectrolytes, carboxylates, phosphonates, polyphosphates, and small peptides, have been shown to either retard or prevent crystallization, thereby extending ACP’s lifetime.^4,40–42^ Additionally, analysis of Zone 4 duration corresponding to the stage where HA formation proceeds until complete ACP transformation, revealed that citrate notably slowed this subsequent stage, whereas BSA had minimal impact (Figure 3). These findings indicate that citrate distinctly influences both the nucleation and growth kinetics of HA during ACP transformation.^43^

## Conclusion

Calcium phosphate precipitation systems are inherently dynamic, and the reaction pathways they follow depend strongly on the specific chemical and physical environment. In this work, it is demonstrated that even under identical final solution compositions, variations in precursor mixing regimes lead to measurable differences in ACP nucleation kinetics and lifetime. In particular, the *P in Ca* mixing pathway resulted in delayed ACP formation and extended Zone 2 and Zone 3 durations, which were correlated with fluctuations in pH and reduced phosphate availability during precursor mixing. These findings indicate that time-resolved solution speciation and transient supersaturation conditions established during mixing play a critical role in governing ACP formation.

The presence of additives further modified the crystallization pathway. Both citrate and BSA increased ACP stability, with citrate showing a more pronounced effect by extending both the ACP lifetime and the subsequent transformation stage. This behavior is attributed to stronger electrostatic interactions and higher effective molar concentration of citrate, supported by zeta potential and thermogravimetric analyses.

Such system-specific responses highlight the need for caution when generalizing mechanistic interpretations across different experimental setups. Advances in analytical techniques with high spatiotemporal resolution have enabled unprecedented insight into the dynamic evolution of ACP and its transformation into crystalline calcium phosphates. However, these tools remain limited in accessibility, often require small sample volumes, and operate within supersaturation and time scale ranges that may not fully replicate natural or industrial conditions. In this context, in situ potentiometric monitoring offer a versatile and more accessible alternative for tracking dynamic solution chemistry during multistep precipitation processes. When combined with complementary solid-phase characterization methods, potentiometric measurements can provide valuable kinetic and mechanistic insights into ACP formation and transformation pathways.

## Supplementary Material



## Notes

Detailed descriptions of calibration of electrodes, and thermodynamic calculations of solution speciation, additional solution speciation data, full characterization spectra, additional kinetic and characterization data (PDF)

## Abbreviations

ACP: amorphous calcium phosphate
HA: hydroxyapatite
NMR: nuclear magnetic resonance
TEM: transmission electron microscopy
SAXS: small-angle X-ray spectroscopy
