# Supplementary material for: Mixing Regime Controlled Kinetics of Amorphous Calcium Phosphate Formation and Transformation
Source: ACS Omega. 2026 Jun 12;11(25):37226–34. doi: 10.1021/acsomega.6c01349 (PMC13325383; doi:10.1021/acsomega.6c01349)
Supplement: Supplementary file 1 [file ao6c01349_si_001.pdf]

Supporting Information

Mixing Regime Controlled Kinetics of Amorphous Calcium Phosphate Formation and Transformation

Özgür Gülmez,[a] Eren Demirbilek[a] and Seniz Ucar*[a,b]

[a] Middle East Technical University, Department of Metallurgical and Materials Engineering, Ankara, Turkiye

[b] Norwegian University of Science and Technology, Department of Chemical Engineering, Trondheim, Norway

A. Calibration of electrodes

A1. Ca-ISE probe calibration

The Ca-ISE probe was calibrated prior to each experiment. For the Ca in P and Equal Mix setups, calibration involved addition of calcium solution to a phosphate-free 50 mM KNO_3 blank solution. The rate and volume of calcium addition during calibration matched those used in the precipitation experiments. In the P in Ca setup, calibration involved adding 40 mL of deionized water into a 500 mL solution containing 50 mM KNO_3 and 4 mM $Ca(NO_3)_2 \cdot 4H_2O$ at a rate of 2 mL/min over 20 min. All calibration experiments were conducted under a N_2 atmosphere, with temperature maintained at 25 °C using a circulating water bath.

Thermodynamic calculations of calcium activity were carried out at 2 min intervals by using ion concentrations and measured solution pH with Visual MINTEQ v3.1 software. Calibration curves were constructed by plotting measured potentials (mV) against the logarithm of calcium ion activity, following the Nernst equation (Equation S1). An example of potentiometric measurements during titration of calcium in a blank solution, and the corresponding calibration curve is given in Figure S1.

$$U = U_0 + \frac{2.303RT}{z_i F} log a_i = U_0 + U_N log a_i \qquad \text{(Eq. S1)}$$

U_0: Standard potential of the measuring chain

R: Gas constant

T: Absolute temperature

z_i: Charge of the measuring ion i (including sign)

F: Faraday constant

a_i: Activity of the measuring ion

U_N: Nernst slope

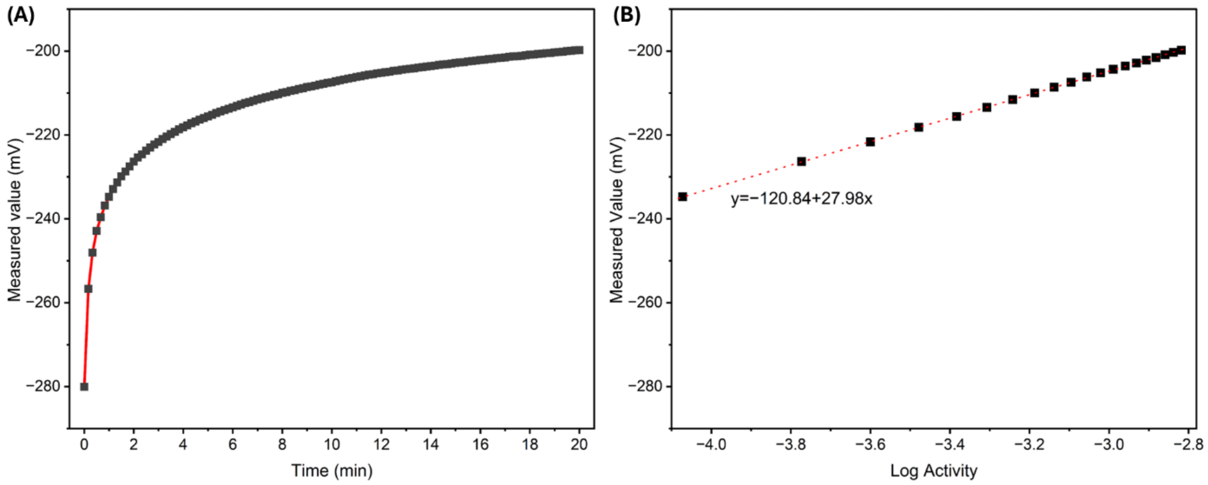

Figure S1. Construction of calibration curve for the determination of Ca^{2+} activity based on the Nernst equation; (A) shows the potentiometric measurements recorded via Ca-ISE as a function of time during titration of calcium solution into 50 mM KNO_3 blank solution, (B) shows the constructed calibration curve with a slope of 27.98 mV, using the experimental measurements on the y-axis vs the calculated calcium activity on the x-axis.

A2. pH probe calibration

pH electrode was calibrated daily. Buffer solutions at pH 4.00, 7.00 and 9.00 were used to cover the relevant pH range.

B. Reactions and their equilibrium constants

The reactions and their corresponding equilibrium constants (-log K) used to calculate solution speciation at 25 °C were obtained from the Minteq v4 database and are listed below.

$H_2O \leftrightarrow H^+ + OH^-$	13.997
$Ca^{2+} + H_2O \leftrightarrow CaOH^+ + H^+$	12.697
$H_3PO_4 \leftrightarrow 3H^+ + PO_4^{3-}$	21.721
H_2PO_4- $\leftrightarrow 2H^+ + PO_4^{3-}$	19.573
$HPO_4^{2-} \leftrightarrow H^+ + PO_4^{3-}$	12.375
$CaH_2PO_4^+ \leftrightarrow Ca^{2+} + 2H^+ + PO_4^{3-}$	20.923
$CaHPO_4 \leftrightarrow Ca^{2+} + H^+ + PO_4^{3-}$	15.035
$CaPO_4^- \leftrightarrow Ca^{2+} + PO_4^{3-}$	6.460
$CaHPO_4.2H_20$ (s) $\leftrightarrow Ca^{2+} + H^+ + PO_4^{3-} + 2H_2O$	18.995
$Ca_5(PO_4)_3OH$ (s) $\leftrightarrow 5Ca^{2+} + 3PO_4^{3-} + H_2O - H^+$	44.333
$Ca_8H_2(PO_4)_6.5H_2O$ (s) $\leftrightarrow 8Ca^{2+} + 6PO_4^{3-} + 5H_2O + 2H^+$	47.950
$Ca_3(PO_4)_2 \leftrightarrow 3Ca^{2+} + 2PO_4^{3-}$ (amorphous 1)	25.500
$Ca_3(PO_4)_2 \leftrightarrow 3Ca^{2+} + 2PO_4^{3-}$ (amorphous 2)	28.250

C. Compositions of precursor and final solutions at the end of mixing

Table S1. The composition of precursor solutions and the final solution after complete mixing for all experimental setups.

Ca in P		P in Ca		Equal Mix			
**Ca-precursor**	**P-precursor**	**Ca-precursor**	**P-precursor**	**Reactor**	**Ca-precursor**	**P-precursor**	**Final solution**
50 mM $Ca(NO_3)_2.4H_2O$	50 mM KNO_3<br>2.4 mM KH_2PO_4<br>1.92 mM KOH	4 mM $Ca(NO_3)_2.4H_2O$<br>50 mM KNO_3	30 mM KH_2PO_4<br>24 mM KOH	50 mM KNO_3	100 mM $Ca(NO_3)_2.4H_2O$	60 mM KH_2PO_4<br>48 mM KOH	50.3 mM K^+<br>53.7 mM NO_3^-<br>3.7 mM Ca^{2+}<br>2.2 mM $PO4^-$
V= 0.04 L	V= 0.5 L	V= 0.5 L	V= 0.04 L	V= 0.5 L	V= 0.02 L	V= 0.02 L	V= 0.54 L

D. Time-resolved pH curves of all independent replicate experiments

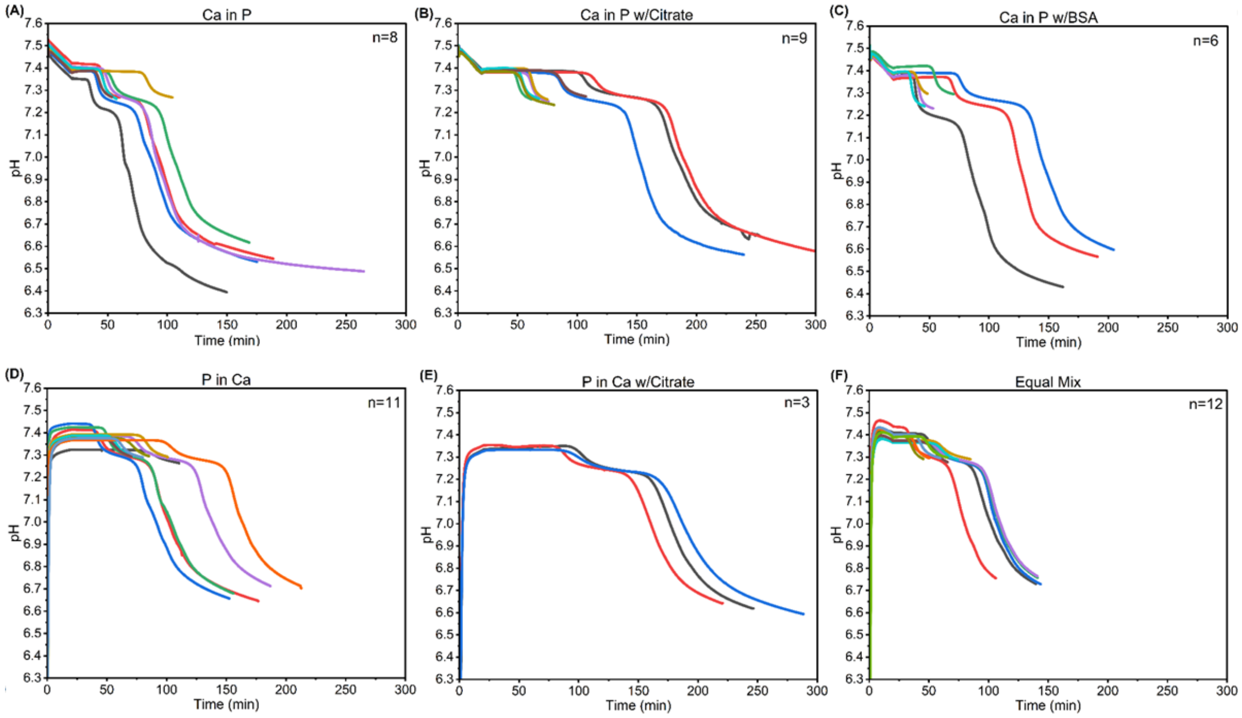

Figure S2. Complete set of pH profiles and experimental replicates recorded during precipitation across all mixing regimes. The panels display the (A-C) *Ca in P* setup (additive-free, with citrate, and with BSA, respectively); (D-E) *P in Ca* setup (additive-free and with citrate, respectively); and (F) *Equal Mix* without additives. Individual replicates are distinguished by different colors, with the replicate numbers (n) indicated in each graph. Note that certain profiles end at Zone 3, representing experiments halted at this stage to collect the intermediate precipitates.

E. Full range FTIR and μ-Raman spectra of precipitates

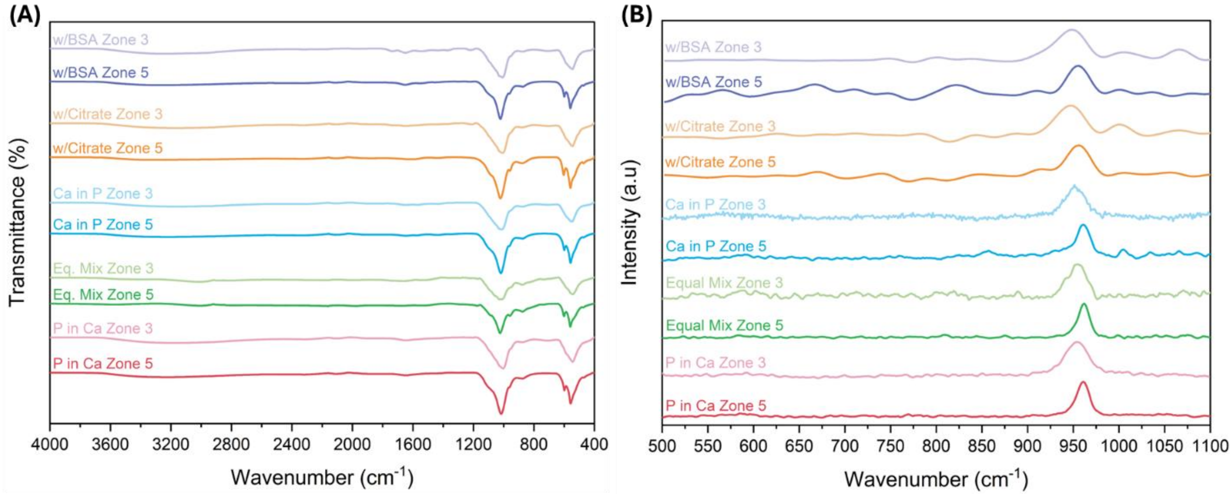

Figure S3. The full range (A) FTIR and (B) μ-Raman spectra of samples collected at indicated time zones for the specified mixing setups and for additive-containing conditions in the *Ca in P* setup.

F. Low angle X-Ray diffraction patterns

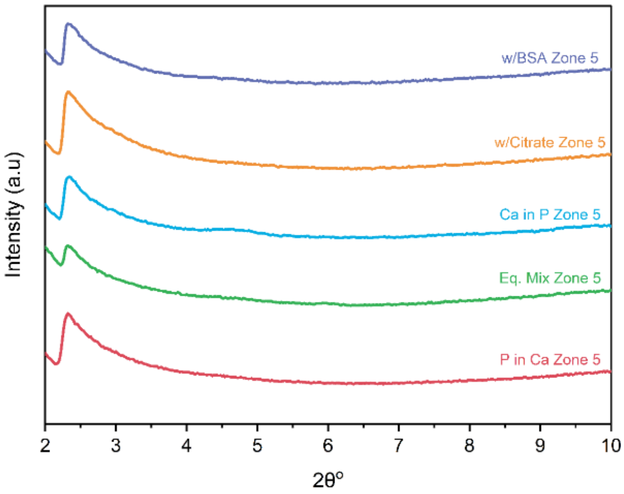

Figure S4. Low-angle X-ray diffraction patterns confirm the absence of octacalcium phosphate (OCP) in the final precipitates for the specified mixing setups and for additive-containing conditions in the *Ca in P* setup, as evidenced by the lack of its characteristic diffraction peak at 4.7° (2θ). The feature observed around 2–2.5° (2θ) is an instrumental artifact arising from the measurement process.

G. SEM and TEM images of precipitates

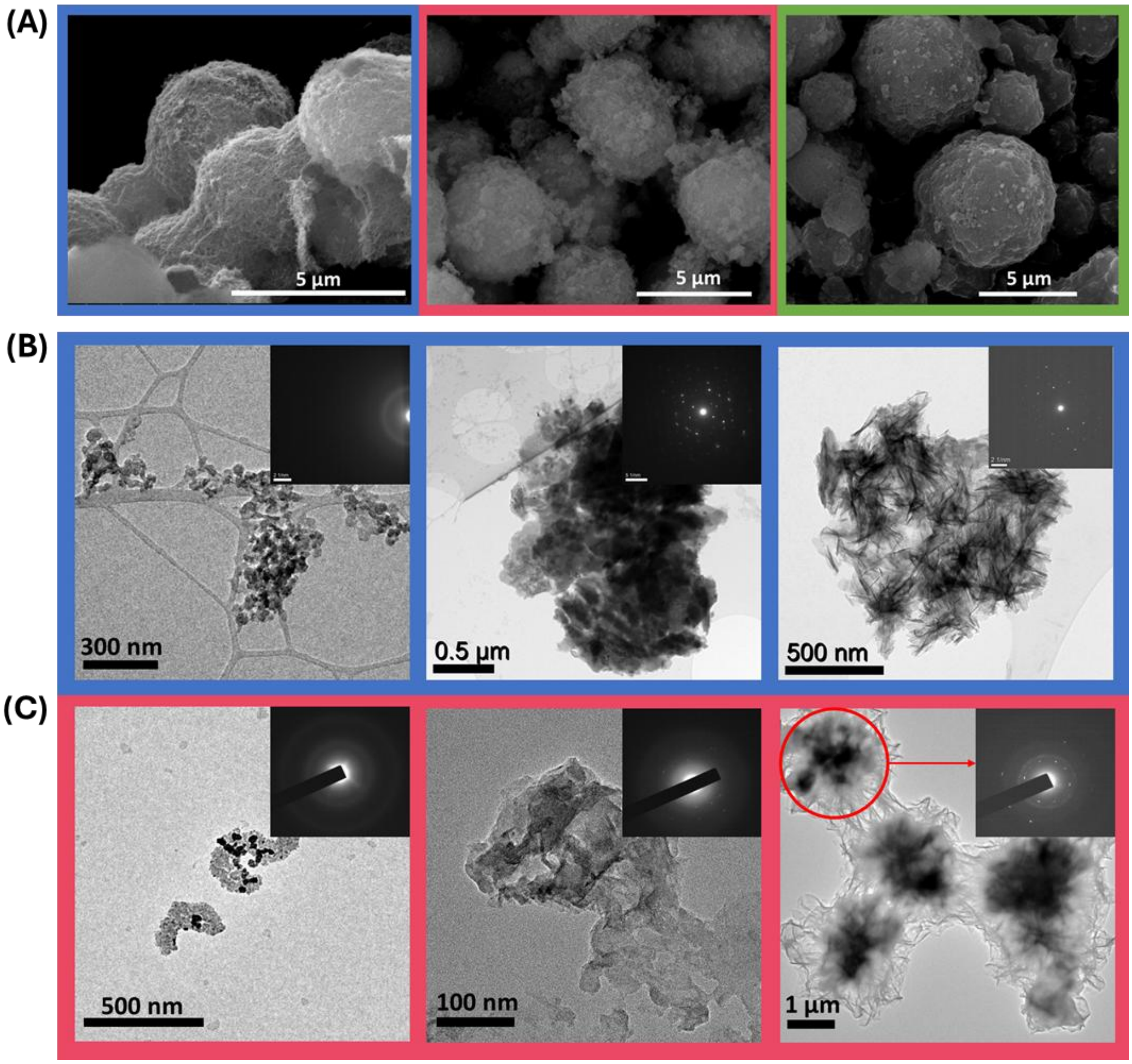

Figure S5. (A) SEM images of samples collected at Zone 3 from the *Ca in P*, *P in Ca*, and *Equal mix* setups (left to right). (B, C) TEM images and corresponding selected area electron diffraction (SAED) patterns of samples collected from the (B) *Ca in P* and (C) *P in Ca* setups at Zones 3, 4, and 5 (left to right). Samples for Zone 4 were specifically extracted during the pH drop between the two plateaus. The evolution of the SAED patterns confirms the gradual phase transformation: Zone 3 exhibits no diffraction spots (characteristic of the amorphous phase), Zone 4 shows the initial emergence of diffraction spots, and Zone 5 displays clear diffraction, confirming the progressive transformation into a poorly crystalline structure.

H. Visual MINTEQ calculations of solution during mixing

H1. Calculation of pH, solution speciation and saturation index

Solution speciation during precursor mixing was calculated using Visual MINTEQ by providing the momentary concentrations of all ions present in the reaction medium at predetermined 1 min intervals, at 25 °C as input. The program determined pH from charge and mass balance, while activity coefficients were calculated using the Davies equation with a *b* parameter of 0.3.

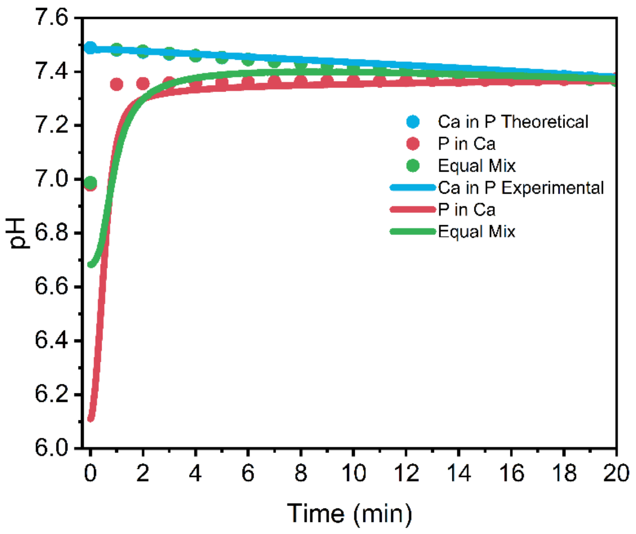

Figure S6. pH evolution as a function of time during the mixing period at indicated additive-free experimental setups Points and lines represent theoretically calculated and experimentally measured values, respectively. Good agreement between calculated and measured pH values is observed for the *Ca in P* setup, attributable to the buffering capacity of the phosphate solution initially present in the reactor. In contrast, larger deviations are observed for the *P in Ca* and *Equal Mix* setups, where the initial solutions lack buffering capacity; the lower experimentally measured pH values in these cases are likely due to CO_2 absorption from the environment. Notably, the *Ca in P* setup exhibits a gradual pH decrease during precursor addition, whereas the other mixing regimes show an initial rapid pH increase followed by stabilization, reflecting their limited buffering capacity. All systems ultimately converge to a similar pH upon completion of mixing.

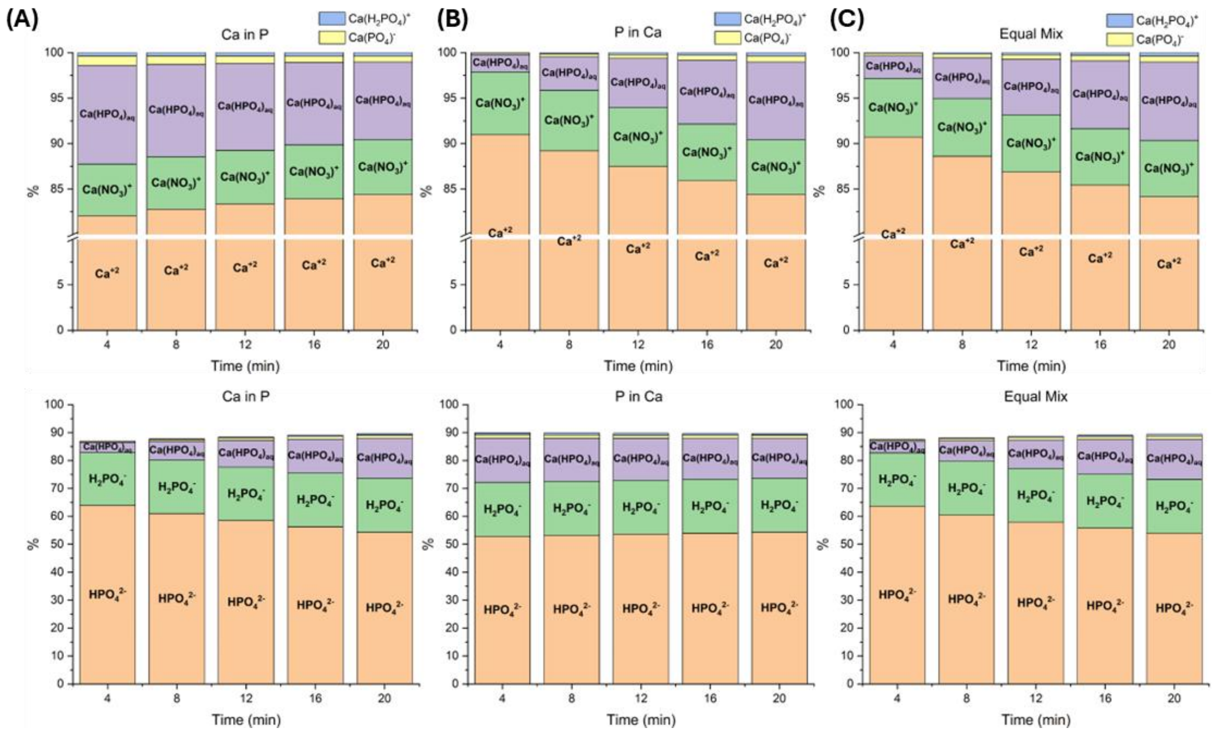

Figure S7. Thermodynamic calculations showing the distribution of calcium and phosphate species during precursor mixing at (A) *Ca in P*, (B) *P in Ca* and (C) *Equal Mix* setups. Note that the species $Ca(H_2PO_4)^+$ and $Ca(PO_4)^-$ are present in trace amounts and appear as thin bands. The phosphate speciation charts (bottom row), sum to approximately 90%, since the remaining ~10% consists of spectator potassium-phosphate complexes (e.g., $KHPO_4^-$, K_2HPO_4(aq), KH_2PO_4(aq)) originating from the precursor salt, and were excluded to highlight only the active calcium-reactive fractions.

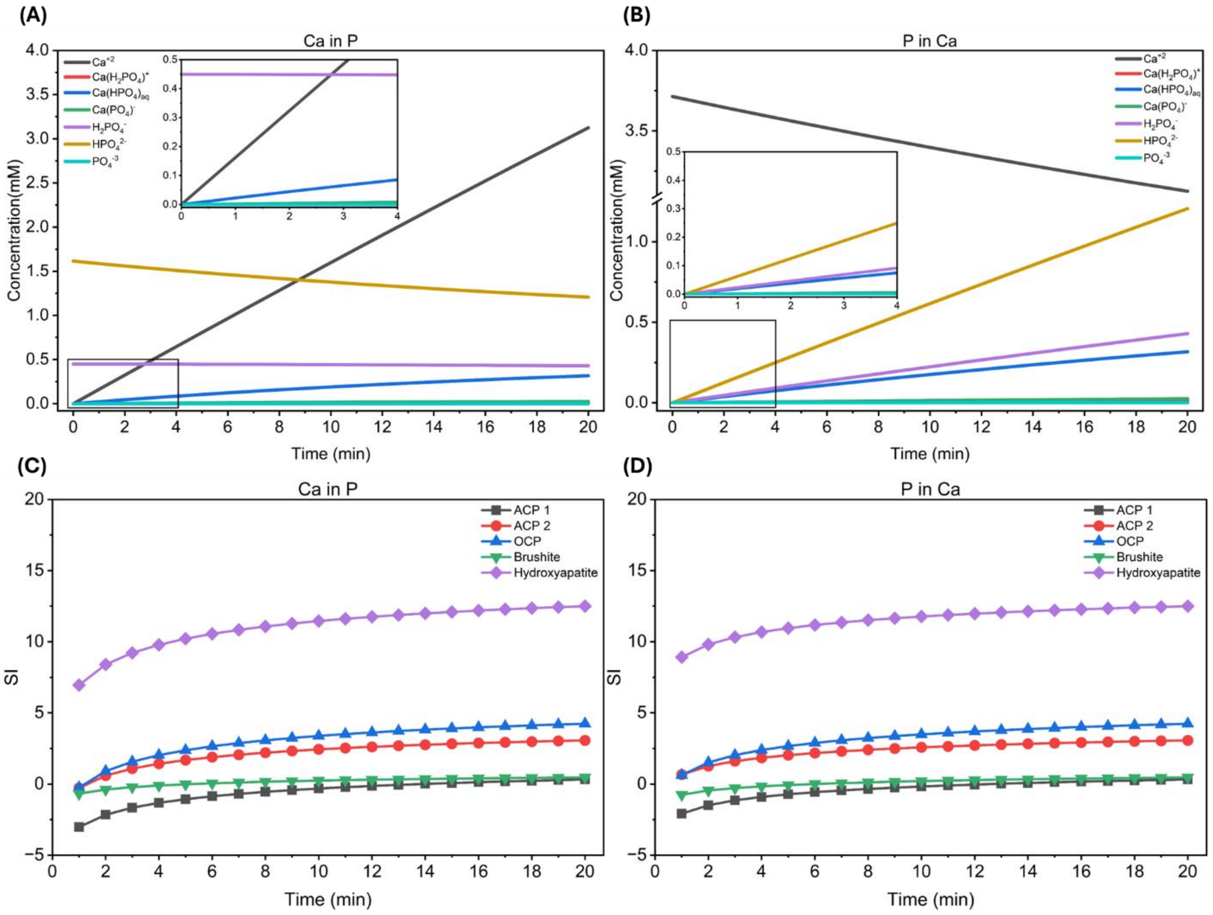

Figure S8. Thermodynamic calculations showing the evolution of solution species concentrations as a function of time during precursor mixing for (A) *Ca in P* and (B) *P in Ca* setups. The corresponding evolution of saturation indices (SI) for ACP, DCPD, OCP, and HA during precursor addition is shown for (C) *Ca in P* and (D) *P in Ca* setups. Saturation indices were calculated according to Equation 1 using solubility constants from the Minteq v4 database. Note:(A)(B) The curves for certain minor species (e.g., $Ca(H_2PO_4)^+$ and $Ca(PO_4)^-$) overlap near the zero baseline due to their extremely low concentrations and may be visually obscured.

H2. Calculation of calcium activity

To quantitatively validate whether the observed delays in amorphous calcium phosphate (ACP) transformation arise from kinetic limitations or thermodynamic constraints (i.e., depletion of free calcium), speciation calculations were performed. Calcium activity during precursor mixing was calculated using Visual MINTEQ by providing the momentary concentrations of all ions present in the reaction medium at predetermined 4 min intervals, at 25 °C as input. Activity coefficients were calculated using the Davies equation with a *b* parameter of 0.3.

Calculations of Calcium Activity in Citrate Presence

To simulate the respective mixing setups containing citrate (e.g., the *Ca in P* setup), 5 ppm citrate was explicitly incorporated into the Visual MINTEQ calculations to evaluate potential Ca^{2+} complexation. The thermodynamic calculations, summarized in Table S2, demonstrate that the addition of 5 ppm citrate has a negligible effect on the bulk supersaturation. During the 20 min

precursor addition period, the decrease in free Ca^{2+} activity reaches maximum ~2% and at 20 min the decrease is as low as 0.6%. Consequently, the Saturation Index (SI) for ACP at the end of the addition phase (20 min) is 3.07 in the additive-free system and 3.06 in the citrate-containing system.

Table S2. Calculated free Ca^{2+} activity and amorphous calcium phosphate (ACP) saturation index (SI) during the precursor addition period (0–20 min) for the *Ca in P* setup, both in the absence and presence of 5 ppm citrate.

	Ca+2 Activity (mM)		SI	
Time(min)	Ca in P	Ca in P w/Cit.	Ca in P	Ca in P w/Cit.
4	0.285	0.278	1.43	1.40
8	0.563	0.554	2.21	2.19
12	0.833	0.824	2.62	2.61
16	1.095	1.086	2.88	2.87
20	1.349	1.341	3.07	3.06

Calculations of Calcium Activity in BSA Presence

Because Bovine Serum Albumin (BSA) is a complex protein absent from standard thermodynamic databases like MINTEQ, its potential impact on free calcium depletion was evaluated stoichiometrically. The molecular weight of BSA is approximately 66.5 kDa. Thus, the molar concentration of a 5 ppm (5 mg/L) BSA solution is calculated as follows:

$$[BSA] = \frac{5 \times 10^{-3}\ g/L}{66500\ g/mol} \approx 0.075\ \mu M$$

Classical equilibrium models establish that a single albumin molecule coordinates with up to 12 calcium ions (Takano et al., 2012). Furthermore, recent molecular dynamics simulations specifically analyzing the Ca-BSA system (Patel et al., 2022) demonstrate that at extreme calcium concentrations, electrostatic binding can theoretically occur at all 97 negatively charged residues on the BSA molecule.

Applying this absolute maximum theoretical binding capacity (97 Ca^{2+} ions per BSA molecule) to our system:

$$Max.Bound\ Ca^{2+} = 97\ \times 0.075\ \mu M = 7.275\ \mu M$$

Considering that the final bulk calcium concentration in our system reaches 3.7 mM (3700 μM), and the maximum possible thermodynamic depletion of free Ca^{2+} by 5 ppm BSA is only ~7.28 μM, this accounts for less than 0.2% of the total available calcium. If the standard coordination limit (12 Ca^{2+} per BSA) is applied, the depletion drops to a mere 0.024%. Therefore, we assume negligible effects of BSA presence on calcium activity, and the calculations were made without correction for BSA binding.

Takano S, Kaji H, Hayashi F, et al. A Calculation Model for Serum Ionized Calcium Based on an Equilibrium Equation for Complexation. *Analytical Chemistry Insights*. 2012;7. DOI:10.4137/ACI.S9681

Patel D, Haag S.L, Patel, J.S et al. Paired Simulations and Experimental Investigations into the Calcium-Dependent Conformation of Albumin. *Journal of Chemical Information and Modeling*. **2022** *62* (5), 1282-1293. DOI: 10.1021/acs.jcim.1c01104

I. pH sensitivity in *P in Ca* setup

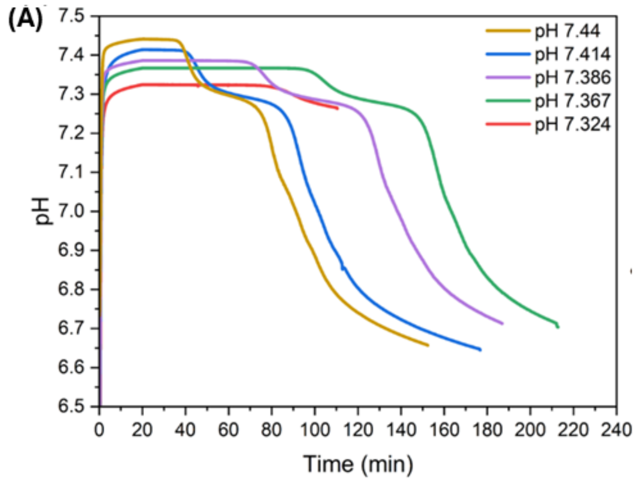

(B)

pH at Zone 2	SI_{ACP}
**7.44**	3.216
**7.41**	3.147
**7.39**	3.101
**7.37**	3.064
**7.32**	2.936

Figure S9. (A) pH profiles from parallel experiments conducted using the *P in Ca* setup, indicating the onset of ACP nucleation, (B) calculated supersaturation indices with respect to ACP, based on precursor solution concentrations and fixed pH values corresponding to those observed experimentally.

J. Precipitation via P in Ca setup with citrate

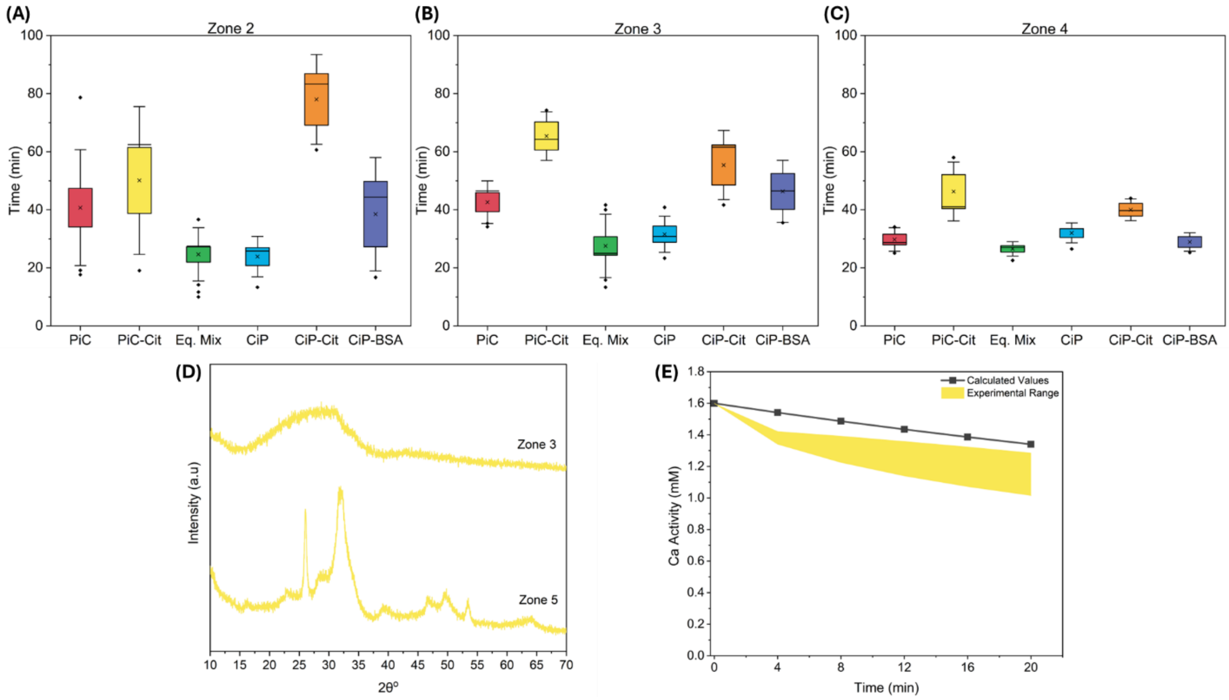

Figure S10. Duration of (A) Zone 2, (B) Zone 3 and (C) Zone 4 under the specified experimental conditions including the data for P in Ca setup with citrate addition, (D) XRD patterns of precipitates collected at specified time zones, (E) Theoretical and measured calcium activities during Zone 1 for the corresponding setup.

K. Determination of Ca/P ratio of ACP phase

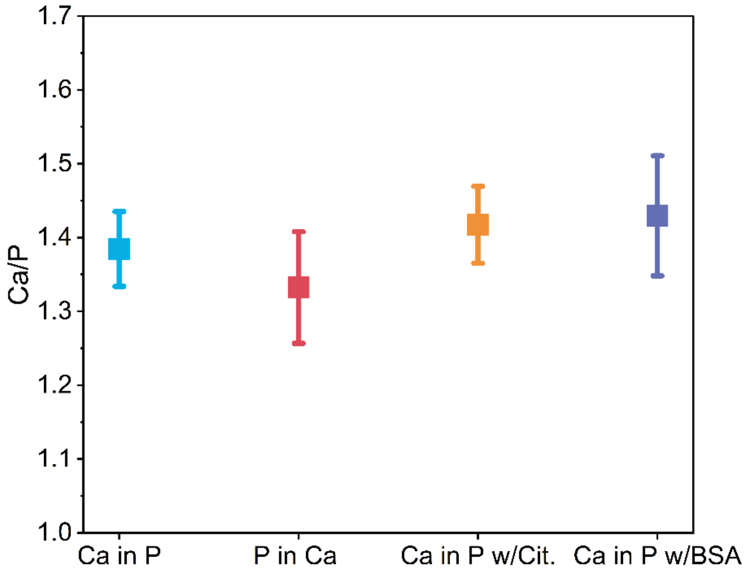

Figure S11. Ca/P molar ratios of ACP precipitates collected from the indicated experimental setups.
